# Powering Molecular Motors with Light Across the Rainbow Using Quantum Dots

**DOI:** 10.1021/jacs.5c05548

**Published:** 2025-09-19

**Authors:** Jiayi Liu, Shuai Zhang, Lin Xi, Jiawei Liu, Yuxin He, Rui Wang, Da-Hui Qu, Ben L. Feringa, Tiegen Liu, Lili Hou

**Affiliations:** † State Key Laboratory of Precision Measurement Technology and Instruments, School of Precision Instruments and Optoelectronics Engineering, 12605 Tianjin University, 92 Weijin Road, Tianjin 300072, China; ‡ Key Laboratory for Advanced Materials and Feringa Nobel Prize Scientist Joint Research Center, School of Chemistry and Molecular Engineering, 47860 East China University of Science and Technology, 130 Meilong Road, Shanghai 200237, China; § Stratingh Institute for Chemistry, University of Groningen, Nijenborgh 4, Groningen 9747AG, the Netherlands

## Abstract

Overcrowded alkene-based molecular motors have high potential in building artificial molecular machines, precise drug delivery, and smart responsive materials due to their unidirectional rotation at the molecular scale under UV light. Powering molecular motors with visible light, especially in the low-energy green, yellow, and red regions, has been a significant challenge, limiting their further application. Herein, we report a general and versatile strategy to overcome this challenge and drive molecular motors using low-energy, low-intensity, noncoherent light across the visible spectrum. Our approach is achieved by simply mixing molecular motors with semiconductor colloidal quantum dots (QDs) and a triplet mediator (9-anthracenecarboxylic acid). The size-tunable absorption of QDs can precisely match the desired color for activating the motor. The mechanism of our design relies on the unique property of QDs, which can efficiently sensitize molecular triplets. Through two-step triplet energy transfers, the rotation of the motor can be efficiently activated using low-energy photons. For the first time, we accomplished driving molecular motors at wavelengths beyond 530 nm under low-intensity and noncoherent light. This breakthrough not only expands the capabilities of visible-light-activated molecular systems to operate in a broad wavelength range but also opens numerous opportunities toward controlling dynamic functions while circumventing competing photochemical processes (i.e., photodegradation).

## Graphical abstract

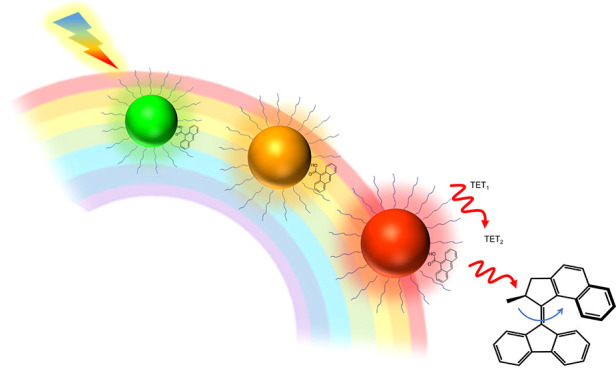

## Introduction

The development of molecular machines has significantly advanced our ability to control motion at the smallest scales.^1,2^ The capabilities of molecular machines can perform many revolutionary tasks by pushing to the scale limit, such as precise drug delivery within organisms and cells, molecular surgery, the creation of nanorobots, and the development of smart responsive materials and devices at the nanoscale,^3–9^ etc. In order to construct a molecular machine, a number of building blocks are required, and at the heart of the rotary function of overcrowded alkene-based molecular motors. These motors can convert light and thermal energy into repetitive 360° unidirectional rotation out of equilibrium.^10^ The rotation occurs via four steps: two UV light-induced *stable/unstable* photoisomerization steps and two irreversible thermal helix inversion (THI) steps.^11^ The helicity of the configuration changes during each step, with the unidirectional rotation determined by the intrinsic chirality of the bulky substituents around the central alkene double bond. Many research efforts in molecular motors have contributed to the design and synthesis of molecular motors with improved efficiency and rotatory speed, as well as to their application in precisely controlling motion at the nano, micro, and macroscales.^12–16^

Using light to power molecular motors offers various advantages, including external and noninvasive control, high spatiotemporal resolution, and ease of tuning wavelengths and intensity. However, most molecular motors rely on the utilization of ultraviolet (UV) light to induce the *stable/unstable* photoisomerization steps.^17,18^ The high energy of UV photons can cause side photoreactions, reducing the motor’s repetitive rotation and degrading the combined materials. Additionally, the short penetration depth of UV light further limits applications in building artificial molecular machines, smart responsive materials, and biological operations. Therefore, developing molecular motors powered by low-energy photons is highly desirable.^19–21^ Many research efforts have been dedicated to this goal. One approach is shifting the absorption band of the motor to the visible region via chemical modifications, such as introducing a large π-conjugation system, “push–pull” substituents, oxindole scaffolds, or salicylidene Schiff base units in the stator and/or rotor halves of the molecules.^22–28^ However, these complex chemical syntheses generally achieve red-shift bands within 100 nm. The longest excitation wavelength reported is 530 nm, located at the red tail of the absorption band, with a very low conversion (<5%) at the photostationary state (PSS).^26^ Another approach is driving molecular motors via the low-lying triplet excited state through triplet energy transfer (TET) from metal complex sensitizers, such as zinc, copper, platinum, ruthenium, and palladium complexes.^29–33^ The longest excitation wavelength for this approach is also around 530 nm, achieved by exciting palladium porphyrin at its weakly absorbed Q-band.^19,33^ The strategy to power molecular motors in the near-infrared region can be achieved through two-photon absorption.^34–36^ However, this strategy requires the use of a highly intense coherent laser source, typically a femtosecond pulsed laser. Therefore, it is highly desireable to develop a simple, efficient, and general approach to drive molecular motors across the entire visible range, especially in the green, yellow, and red regions, using a noncoherent, low-intensity light source.

Herein, we report a general strategy for powering molecular motors using low-energy, low-intensity, noncoherent light across the entire visible spectrum through a two-step TET process, using QDs as sensitizers and 9-anthracenecarboxylic acid (9-ACA) as a mediator (Figure 1). The wavelength of the powering light is adjustable by matching the color with appropriately sized QDs, as shown in Figure 1b. This is achieved by utilizing the features of QDs: tunable sizes and bandgaps, strong absorptivity, as well as the ability to efficiently sensitize molecular triplets.^37,38^ The mechanism of our design is illustrated in Figure 1c. Visible light irradiation on QDs populates the “dark” triplet state of surface-anchored 9-ACA via the first triplet energy transfer (TET_1_) step. Subsequently, the long-lived triplet 9-ACA transfers energy to the molecular motor through the TET_2_ step, activating *stable/unstable* photoisomerization along the low-lying triplet reaction pathway. Though the triplet energy of 9-ACA is slightly lower than that of the motor, TET can still occur under endergonic conditions.^39^ Three different-sized CdSe QDs were prepared to match the absorption windows of green, yellow, and red light, for the first time enabling the activation of molecular motors at wavelengths longer than 530 nm using low-intensity, noncoherent light sources.

**Figure 1:**
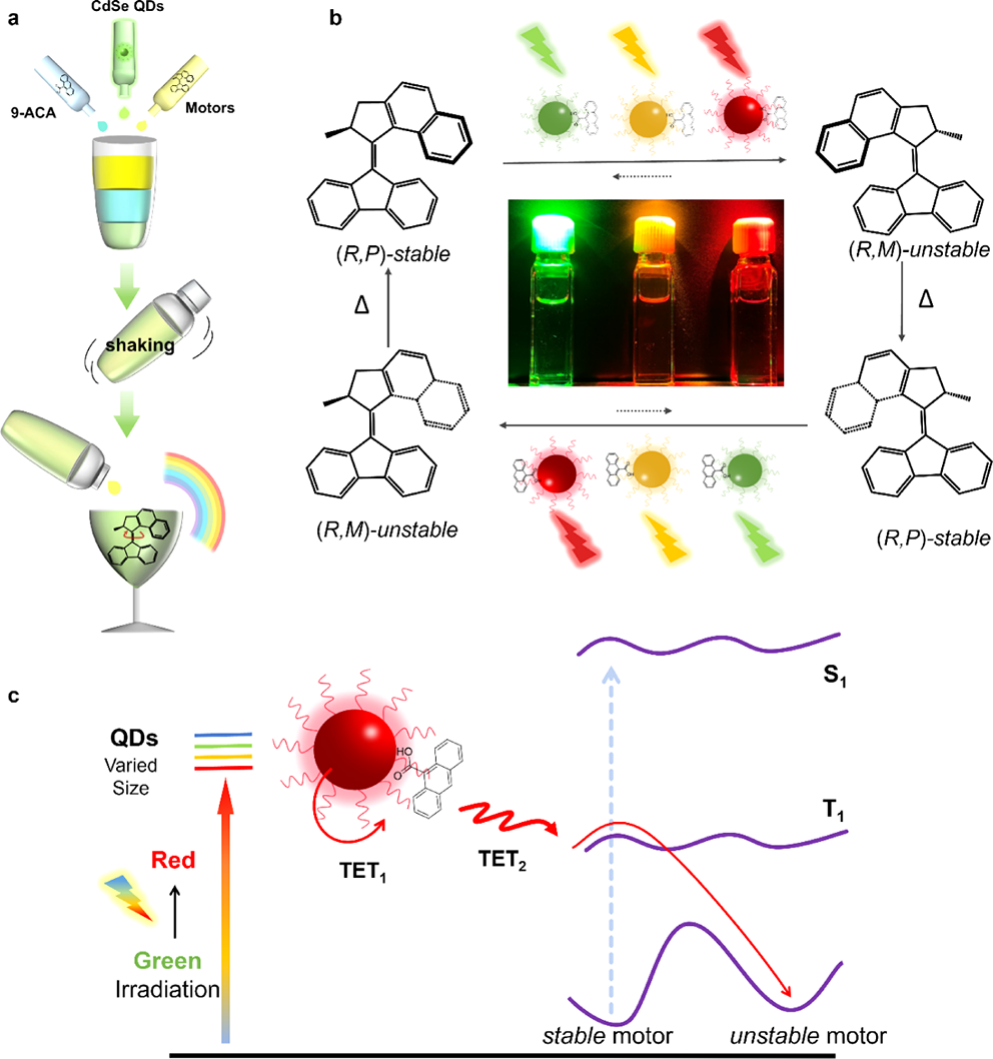
Powering molecular motors under the rainbow using quantum dots (QDs). (a) Preparation process of the “cocktail” solution by mixing QDs, 9-ACA, and the motor for visible-light activation. (b) Powering molecular motor rotation using light across the rainbow spectrum by introducing variously sized QDs. (c) Mechanism of motor activation through two-step TETs upon visible light irradiation on QDs. The blue arrow indicates direct activation through the singlet excited state using UV or blue light. The red arrows indicate activation through TET in the “cocktail”.

## Results and Discussion

### Design and Characteristics of QD Synthesis

To match the desired light color to activate the motor—specifically green (530 nm), yellow (590 nm), and red (630 nm) light—CdSe QDs were chosen due to their appropriate absorption bands. The position of the absorbance band of QDs is highly correlated with particle size^40^ which can be precisely controlled by adjusting the reaction time and temperature.^41,42^ CdSe QDs were synthesized according to previous reports.^37^ To achieve the desired particle sizes, the selenium precursor injection temperature and reaction time were carefully optimized, with detailed information provided in the Supporting Information. Three batches of QDs, named CdSe 560, CdSe 590, and CdSe 630, were obtained, and their UV–visible absorption spectra are shown in Figure 2a, represented by the colors green, yellow, and red, respectively. The first absorption bands of QDs are located in the desired light irradiation region, with the peaks in toluene at 560, 590, and 630 nm, respectively. The diameters of QDs, estimated from the first absorption peak^43^ are 3.5, 4.2, and 6.2 nm, consistent with the observations in TEM images of QDs (Figure S1). These QDs exhibit distinct luminescence colors, with the photoluminescence (PL) peaks located at 573, 610, and 640 nm, as shown in Figure 2b. The detailed photophysical properties of the three batches of QDs are summarized in Table S1.

**Figure 2:**
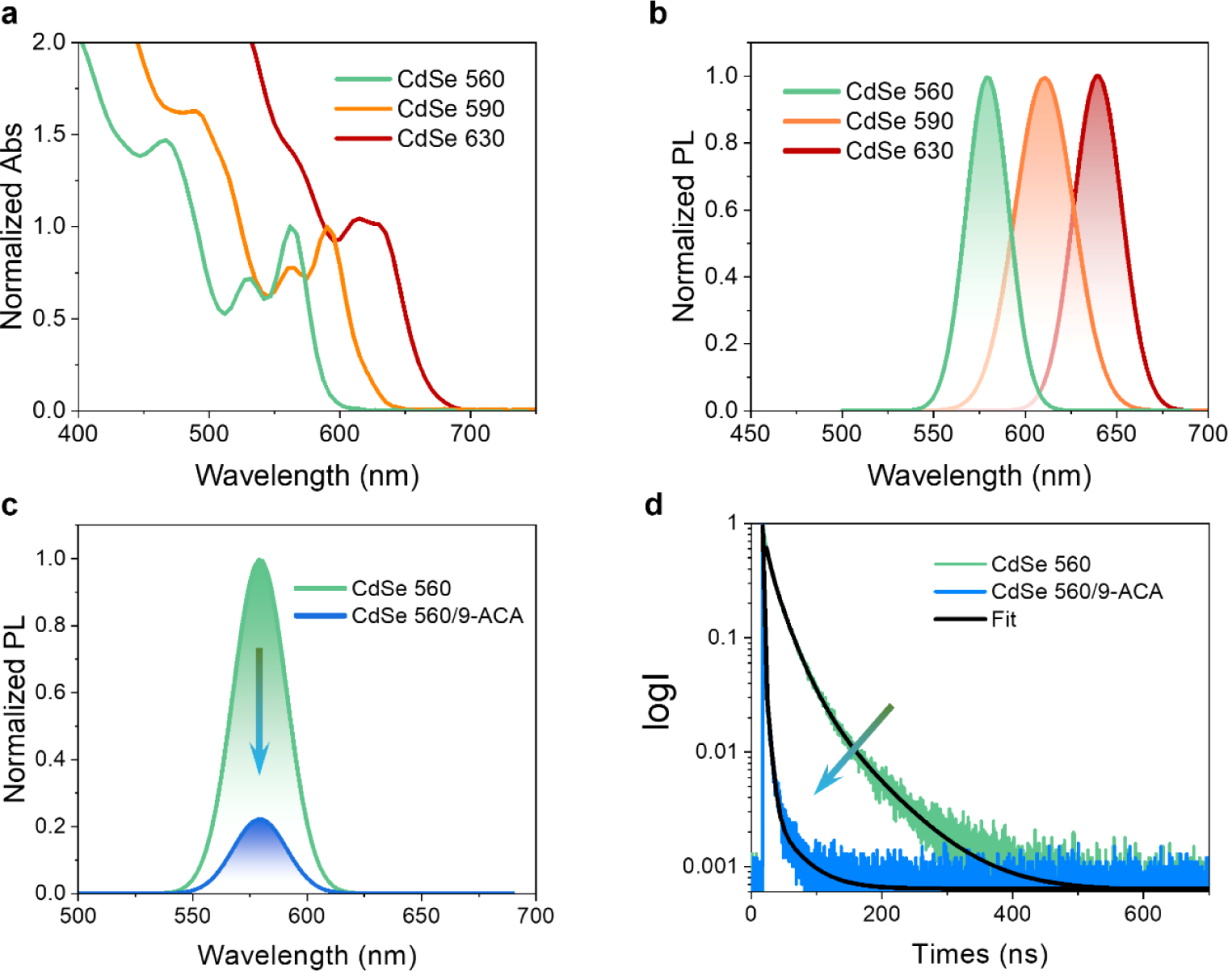
Photophysical properties of QDs and PL quenching after mixing with 9-ACA. (a) UV–visible absorption and (b) PL spectra of CdSe 560, CdSe 590, and CdSe 630 in toluene. The spectra are normalized at the first absorption peak of QDs and maximum PL intensity. (c) PL spectra of 3.1 μM CdSe 560 before and after mixing with 0.2 mM 9-ACA in toluene. (d) PL lifetimes and fitting of CdSe 560 before and after the addition of 9-ACA.

Due to the short-lived triplet excited state (*T*_1_) of QDs, a mediator anchored to the surface of the QDs is required to harvest the QD’s triplet and yield a long-lived molecular triplet state,^37,44^ which can then activate molecular motors. To enable the relay of TETs between QDs and molecular motors, energetic matching of the triplet states is necessary; that is, the *T*_1_ energy of the mediator should be positioned between those of the QDs and molecular motors. Due to strong spin–orbit coupling, QDs exhibit substantial mixing of the singlet and triplet states,^45^ and both the bright and dark exciton states can contribute to TET.^46^ In our case, the *T*_1_ energies of CdSe 560, CdSe 590, and CdSe 630 can be estimated from the peak of the first excitonic transition to be 2.31, 2.08, and 1.94 eV, respectively. Considering the *T*_1_ energy of the molecular motor is 1.89 eV,^33^ 9-ACA, with a similar triplet energy (1.83 eV), was chosen as the mediator,^47^ and the absorption and PL spectra of 9-ACA are shown in Figure S2. The carboxylic acid group of 9-ACA facilitates its adsorption onto the surface of the QDs, thereby efficiently harvesting the QDs’ triplets via Dexter-type TET.^37^

A simple, direct mixing approach is employed to prepare the QDs/9-ACA hybrid, and our previous works indicate that this noncovalent method can yield efficient TET through dynamic collisions.^48,49^ The concentrations of each component in the mixture were optimized by titration while monitoring changes in the PL (see details in the Sample Preparation section of the Supporting Information). When mixing CdSe QDs with 9-ACA in toluene, the absorption features of the QDs and 9-ACA remain unchanged, as shown in Figure S3, indicating no ground-state interaction between them. In contrast, significant quenching in the PL spectra was observed in all three QDs (Figures 2c and S4a,b), attributed to the efficient TET_1_ from QDs to 9-ACA via the excited state, which is consistent with previous reports.^37,50^ The quenching efficiencies estimated from PL intensity quenching are 80.6% for CdSe 560, 73.2% for CdSe 590, and 85.2% for CdSe 630, respectively. The PL lifetimes of QDs before and after mixing with 9-ACA were also determined, as shown in Figures 2d,S4c,d, and Table S2. The lifetimes of QDs reduce from 29.3 to 4.2 ns for CdSe 560, from 28.3 to 7.2 ns for CdSe 590, and from 18.5 to 2.3 ns for CdSe 630, respectively. The efficiencies estimated from PL lifetime are 85.6%, 75.0%, and 87.5%, respectively. The decreased quenching efficiency from CdSe 560 to CdSe 590 can be attributed to the reduced driving force for the TET_1_ step (from 0.48 eV to 0.25 eV). The reduction in the PL lifetime further demonstrates the TET_1_ process in all three cases. The significant quenching observed for CdSe 630 may be due to the formation of nonradiative trap centers, resulting from the relatively lower quality of the nanocrystals, as evidenced by their reduced PLQY (Table S1).

### Activation of Molecular Motors under Colorful Visible Light

A typical second-generation molecular motor (Figure 1b) was chosen to confirm the visible light activation of our design, and the motor was synthesized according to previous reports.^51^ Under 365 nm UV light irradiation (5 mW, 3 min, RT 25 °C), the UV–visible absorption spectrum of the motor shows a redshift from the peak maximum of 395 to 425 nm (Figure S6a), indicating the *stable/unstable* photoisomerization steps of the motor’s rotation via the singlet excited state (*S*_1_). Remaining in the dark for 20 min at RT, full recovery of the absorption spectra was observed, indicating the THI steps of motor rotation. The presence of an isosbestic point at 408 nm in both the photoisomerization and THI steps demonstrates a unimolecular reaction (Figure S6b). Directly irradiating molecular motors under 550, 590, or 635 nm light reveals no changes in the UV–visible absorption (Figure S7), as the energy of these photons is insufficient to access the singlet excited state of the motor to activate the rotation.

To demonstrate the visible light activation of our design, the sample was prepared using a simple “cocktail” approach (Figure 1a): first by adding 9-ACA to the QDs solution, followed by the addition of the molecular motor solution. All samples were prepared in a glovebox to ensure an oxygen-free environment for the TET processes. Detailed information regarding sample preparation is available in the Supporting Information. Under irradiation from a 550 nm LED light source (140 mW/cm^2^, 3 min), the mixed solution of CdSe 560 (3.1 μM), 9-ACA (0.2 mM), and molecular motors (0.14 mM) exhibits a red shift in the range from 400 to 500 nm, as shown in Figure 3a, with an isosbestic point at 408 nm (Figure S8). This change is consistent with that observed in the absorption spectra of molecular motors under direct 365 nm light irradiation, indicating that the photoisomerization step is validated upon green light irradiation. The conversion ratio between *stable* and *unstable* motors, calculated from the absorption spectrum, is 67:33, which is very similar to previous approaches using metal complexes to sensitize molecular motors (64:36).^29^ The THI step of the motors undergoes the same process in the mixed solution as it does alone, as evidenced by the recovery of the absorption spectra after leaving the sample in the dark for 20 min at RT. The THI speed of molecular motors in the mixture is also very similar to that in the absence of QDs and 9-ACA, as shown in Figure S9. The visible light activation of the motor is fully repeatable. With three cycles of green light irradiation for 3 min followed by leaving the sample in the dark for 20 min, the change in absorbance at 450 nm exhibits excellent reversibility, as shown in Figure 3d. The repeatable operation of our approach indicates good fatigue resistance without motor degradation under visible light control. To further explore the visible light activation of the motor across the rainbow spectrum, CdSe 590 and CdSe 630 QDs were employed for yellow and red light irradiation. The samples were prepared by the same cocktail approach as for CdSe 560. Figure 3b,c shows similar red shift in the absorption band under 590 nm (101 mW/cm^2^, 3 min) and 635 nm (280 mW/cm^2^, 3 min) LED light source irradiation, and these changes can fully recover in the dark via the THI process. The repeatable operations of the motor in Figure 3e,f indicates that yellow and red light can successfully drive molecular motors when utilizing appropriately sized QDs.

**Figure 3:**
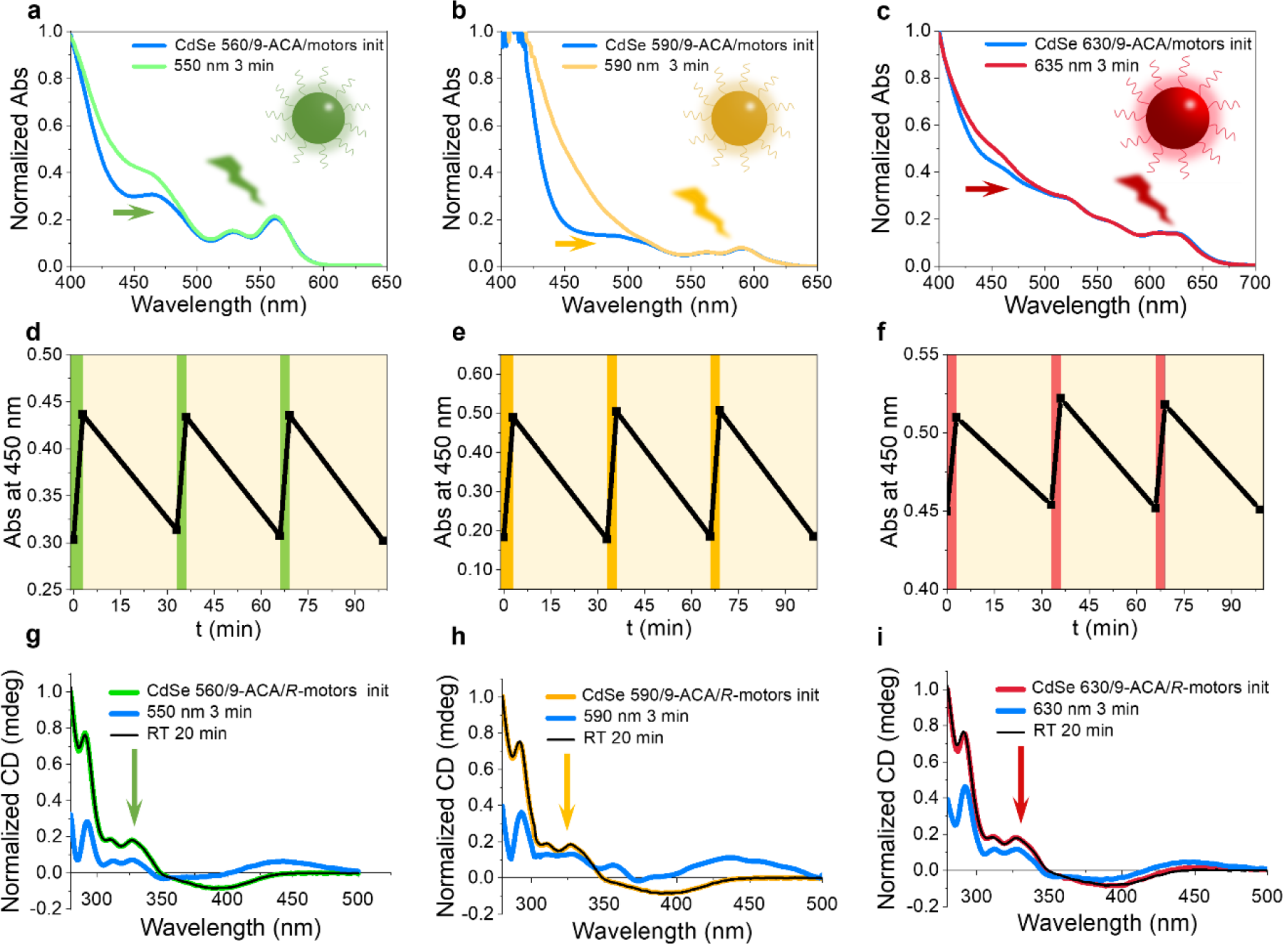
Absorption and CD spectra of the QDs/9-ACA/motor cocktail solution before and after visible light irradiation. Absorption spectra of the mixture irradiated with (a) 550 nm (140 mW/cm^2^), (b) 590 nm (101 mW/cm^2^), and (c) 635 nm (280 mW/cm^2^) LED light sources for 3 min. Repeatability of motor rotation, as monitored by the absorbance at 450 nm over 3 cycles of first irradiation at (d) 550 nm, (e) 590 nm, and (f) 635 nm light, followed by relaxation during the thermal step in the dark at RT. CD spectra of enantiomerically pure *R*-motors mixed with (g) CdSe 560, (h) CdSe 590, (i) CdSe 630, and 9-ACA after 3 min of visible irradiation and after the thermal step for 20 min.

It should be noted that, in the absence of 9-ACA in the cocktail solutions, the motor cannot rotate under visible light irradiation (Figure S10) because the triplet lifetime of CdSe QDs is too short to directly sensitize the motor’s triplet state. Our approach of using QDs to activate the rotation of molecular motors under noncoherent visible light irradiation is not limited to CdSe QDs but it is also versatile enough to apply to other types of QDs, such as CdTe QDs. We have synthesized CdTe QDs, named CdTe 620, with the first absorption peak around 620 nm, as shown in Figure 4a. When cocktailed with 9-ACA and the motor, the rotation can be observed under 635 nm LED light source irradiation, as shown in Figure 4b, and the conversion ratio between *stable* and *unstable* was determined to be 77:23.

**Figure 4:**
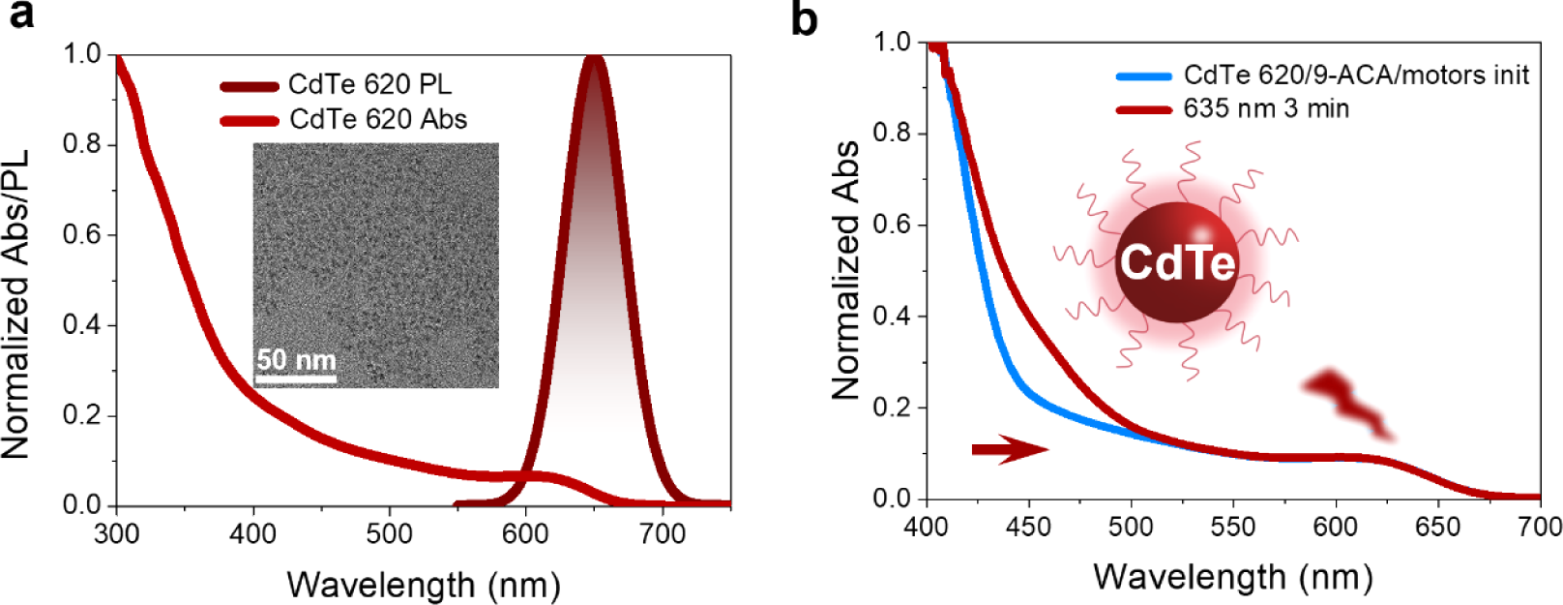
CdTe QDs were employed to drive molecular motors under red light irradiation. (a) Normalized UV–visible absorption and PL spectra of CdTe 620. (b) Absorption spectra of a CdTe 620/9-ACA/motor mixture before and after irradiation at 635 nm (3 min, RT).

The rotation of molecular motors under visible light is further confirmed by Circular Dichroism (CD) spectroscopy. Enantiomerically pure motor samples (*S, R*) were obtained by chiral stationary phase HPLC. The *R*-motor exhibits a positive Cotton effect^17^ located at 280–350 nm and a negative band in the region between 350 and 450 nm in toluene (Figure S11a). The chiral nature of the motor is retained when mixed with CdSe 560 and 9-ACA, as shown in Figure 3g. Upon 550 nm green light irradiation, both the positive and negative bands in the CD spectrum of the mixture decrease in intensity, and the formation of a new positive band was observed between 410 and 500 nm, indicating the formation of *unstable* isomers with opposite helicity. After leaving the mixture in the dark at RT for the THI step, the CD spectrum fully recovers to the initial state. The same changes are observed in the enantiomerically pure *S*-motor mixture, with an opposing signal in the CD spectra (Figure S12a). When enantiomerically pure motors are mixed with CdSe 590 or CdSe 630, under 590 nm yellow or 635 nm red light irradiation, the rotation of the motors induces similar changes in the CD spectra (Figures 3h,i and S12b,c).

The efficiency of our visible light activation of molecular motors was further investigated, and the photochemical properties are summarized in Table S3. The conversion ratios between *stable* and *unstable* states at PSS were determined to be 67:33 for 550 nm irradiation, 69:31 for 590 nm irradiation, and 80:20 for 635 nm irradiation, respectively; see the detailed determination described in the Supporting Information. We notice that our approach using green and yellow light activation achieves a higher conversion than Pd-porphyrin-sensitized molecular motors via noncovalent control (532 nm, 75:25) and is nearly as efficient as Pd-porphyrin covalently linked to the motor (532 nm, 67:33).^33^ Our efficient cocktail approach to driving the motor with visible light is due to the high energy transfer efficiency and long-lived triplet state of 9-ACA (*vide infra)*. In order to further evaluate the efficiency of our visible light activation approach, we determined the photochemical quantum yields (QYs) of our design at three visible light irradiation wavelengths; see the Supporting Information for determination details. The QYs are 3.8% at 550 nm, 3.3% at 590 nm, and 2.1% at 620 nm, respectively, which are similar to those of the push–pull motor under 455 nm light irradiation.^26^ It should be noted that the photochemical QY of the visible-light-activated motor is positively correlated with the photoluminescence quantum yield (PLQY) of CdSe QDs and negatively correlated with the size of the QDs. The photochemical QY and motor conversion decrease with red light activation because the larger CdSe QDs feature more defects, as indicated by their low PLQY, leading to excited energy dissipation through nonradiative decay channels. Additionally, the narrower bandgap between CdSe 630 (1.94 eV) and 9-ACA (1.83 eV) reduces the driving force for the stepped energy transfer.^44,47,52^ Therefore, although high photoquenching efficiency and quenching rate constants were observed in the case of CdSe 630, motor activation results indicate that nonradiative trapping and relaxation compete with TET,^53^ thereby reducing the efficiency of sensitized isomerization. Further improving the quality of QDs via optimizing QD synthesis or by introducing other QDs with fewer defects in the red region, to minimize the competition between defect states and 9-ACA for TET, can potentially enhance the red-light conversion of the motor. Nevertheless, the conversion ratio at PSS under red-light activation significantly surpasses that of the push–pull substituted motor with green-light activation (530 nm, 95:5).^26^ It should be noted that our approach is also the first example to realize driving molecular motors using weak, noncoherent yellow and red light.

### Mechanism Investigation

To gain further insight into the mechanism of our approach using QDs to activate molecular motors across the rainbow spectrum, femtosecond transient absorption (fsTA) and nanosecond transient absorption (nsTA) spectroscopy measurements were carried out. For free CdSe 560, the long-lived decays in the fsTA spectra (Figure 5a) are associated with the ground-state bleach (GSB, >500 nm) and excited-state absorption (ESA, 390–500 nm),^37,54^ showing 52% decay within a time window of 6 ns. When inducing 9-ACA, accelerated decays occur in both the GSB and ESA of CdSe 560, as shown in Figure 5b. Meanwhile, the emergence of a positive absorption band at 430 nm is observed due to the transition of *T*_1_ to *T_n_* in 9-ACA, confirming the formation of triplet 9-ACA by TET from CdSe QDs. The rate constant for TET can be estimated by fitting the time-resolved trace at 506 nm (Figure 5c), resulting in *k*_TET506_ = 1.5 × 10^9^ s^–1^, which is nearly identical to that estimated from triplet formation of 9-ACA at 430 nm (*k*_TET430_ = 1.2 × 10^9^ s^–1^) (Figure 5b inset). Details regarding the fitting and rate constant calculation procedures are found in the Supporting Information. The nsTA spectroscopy measurements were employed to investigate the energy transfer on longer time scales. Free CdSe QDs show no obvious nsTA feature at 2 μs (Figure S13a). When inducing 9-ACA to CdSe 560, the formation of triplet 9-ACA via the TET_1_ step can be evidenced by the appearance of a *T*_1_ to *T_n_* transition band centered around 430 nm, as shown in Figure 5d, which is consistent with previous reports.^37,55,56^ The triplet lifetime (*τ*_0_) of 9-ACA was determined to be 267 μs by fitting the time-resolved TA decay at 430 nm (inset in Figure 5d). The formation of triplet 9-ACA was also evidenced in the cases of CdSe 590 and CdSe 630 (Figure S13b,c), and the generated triplet 9-ACA exhibits lifetimes of 227 and 5.1 μs, respectively. The short *τ*_0_ of 9-ACA when mixed with CdSe 630 results from the greater number of defects in the larger QDs and the decrease in efficiency of charge and energy transfer,^57^ which explains the low conversion to the *unstable* motor when irradiated under red light. Further cocktailing with the motor sample results in a decrease in the triplet lifetime of 9-ACA, as shown in Figure 5e, because of the TET_2_ step from triplet 9-ACA to the triplet motor. Figure 5f shows the quenched triplet lifetime ratio of 9-ACA as a function of the motor concentration. The fitting of the Stern–Volmer equation, *τ*_0_/*τ* = 1 + *τ*_0_ *k*_*q*_[*Q*] (where, *τ* is the triplet lifetime of 9-ACA with the motor and [*Q*] is the concentration of the motor), gives the bimolecular quenching rate constant *k_q_* = 1.4 × 10^8^ M^–1^ s^–1^, indicating that the TET_2_ step is under diffusion controlled. The slow quenching rates can be attributed to the endergonic TET, as the triplet energy level of 9-ACA is lower than that of the motor. The quenching of triplet 9-ACA by the motor was also measured in combination with CdSe 590 and CdSe 630; see the details in Figure S14. It is interesting to note that quenching rate constants increased in these two cases, with *k*_*q*_ of 3 × 10^8^ M^–1^ s^–1^ and 3.5 × 10^9^ M^–1^ s^–1^, respectively. The increased *k*_*q*_ is possibly due to the larger surface area-to-volume ratio, which enhances the probability of intermolecular interactions between 9-ACA and molecular motors. Additional defect-induced nonradiative pathways may also contribute to the high quenching rate constants observed for yellow and red CdSe QDs.

**Figure 5:**
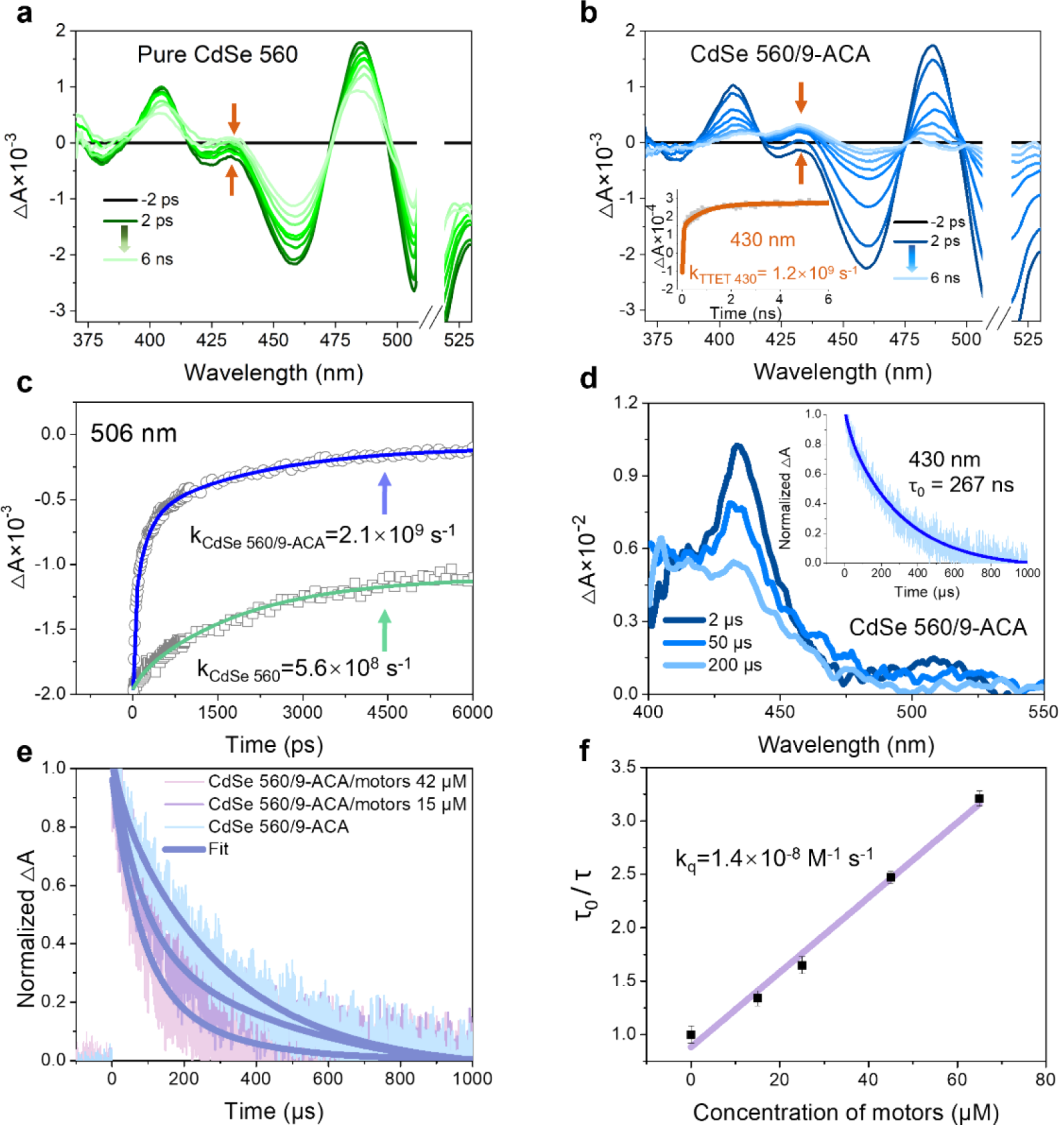
fsTA and nsTA spectroscopies investigated the mechanism of the visible-light-activated motor using QDs. The fsTA spectra of CdSe 560 in the absence (a) and presence (b) of 9-ACA excited at 550 nm. The inset in (b) shows a kinetic analysis of triplet 9-ACA generation at 430 nm. (c) Ground-state recovery process of CdSe 560 at 506 nm before and after the induction of 9-ACA. (d) nsTA spectra of CdSe 560/9-ACA and the time-resolved TA kinetics of the transient signals at 430 nm (inset). (e) Time-resolved TA kinetics of CdSe560/9-ACA at 430 nm when mixed with the motor. (f) Stern–Volmer plot and linear fitting of the triplet lifetime quenching of 9-ACA by the motor.

## Conclusions

In summary, we have demonstrated, for the first time, that the unidirectional rotation of the molecular motor can be activated under weak, noncoherent visible light at wavelengths exceeding 550 nm by introducing QDs as triplet sensitizers. By precisely adjusting the absorption spectra of the QDs, we can fine-tune the driving light to any desired wavelength across the rainbow spectrum. Our design is achieved via a straightforward approach that involves efficient TET transfer from QDs to the surface-anchored 9-ACA, which further enables the second diffusion-assisted TET step from 9-ACA to the molecular motors through dynamic interactions in solution. The efficient and repeatable visible-light-induced unidirectional rotation was confirmed by UV–visible and CD spectroscopies. The uniqueness of our design lies in the ability of QDs to rapidly and efficiently sensitize the triplet states of 9-ACA and subsequently activate molecular motors. Through two consecutive TET steps, rotation of the motor occurs along the low-lying triplet state pathway, as evidenced in the PL and TA spectra. It should be noted that our approach is broadly applicable to other derivative molecular motors by simply adjusting the QDs’ absorption spectra through size variation and matching the triplet energies of the mediator and motor. Additionally, introducing molecular motors with carboxylic acid functional groups to anchor directly to the QDs’ surface, the subject of our future studies, could eliminate the use of a mediator and potentially extend the activation wavelength region. Our simple yet effective approach to activating molecular motors at any desired visible light region paves the way for further developments in molecular machines and smart responsive materials.

## Supplementary Material



## Notes

Synthesis of QDs, sample preparation, spectroscopy, PLQY measurements, photoluminescence lifetime of QDs, kinetics fitting model, photochemical experiments, and supplementary figures (PDF)
