# Supplementary material for: Powering Molecular Motors with Light Across the Rainbow Using Quantum Dots
Source: J Am Chem Soc. 2025 Sep 19;147(39):35255–63. doi: 10.1021/jacs.5c05548 (PMC12498390; doi:10.1021/jacs.5c05548)
Supplement: Supplementary file 1 [file ja5c05548_si_001.pdf]

Supplementary Information

Powering Molecular Motors with the Light across the Rainbow using Quantum Dots

Jiayi Liu[1], Shuai Zhang[1], Lin Xi[1], Jiawei Liu[1], Yuxin He[1], Rui Wang[2], Da-Hui Qu[2*], Ben L. Feringa[1,2,3*], Tiegen Liu[1], Lili Hou[1*]

1. State Key Laboratory of Precision Measurement Technology and Instruments, School of Precision Instruments and Optoelectronics Engineering, Tianjin University, 92 Weijin Road Tianjin, 300072, China;

2. Key Laboratory for Advanced Materials and Feringa Nobel Prize Scientist Joint Research Center, School of Chemistry and Molecular Engineering, East China University of Science and Technology, 130 Meilong Road, Shanghai, 200237, China;

3. Stratingh Institute for Chemistry, University of Groningen, Nijenborgh 4, 9747AG Groningen, The Netherlands.

Methods

1 Chemicals

Selenide (99.999%), tellurium (99.999%), 1-octadecene (ODE, 90%), cadmium oxide (CdO, 99.99%), oleic acid (OA, 90%), tri-*n*-octylphosphine (TOP, 97%), tributylphosphine (TBP, 97%) and *n*-octadecylphosphonic acid (ODPA, 98%) were purchased from Shanghai Aladdin Chemical Reagent Co., Ltd, China. Acetone, acetonitrile and toluene were purchased from YuanLi, Tianjin. All the chemicals were used without further purification steps.

2 Synthesis of QDs

CdSe: The synthesis of CdSe QDs was referred to previous reports[1]. TOP (4.0 mL), ODPA (0.28 g), and CdO (0.06 g) were mixed in a 100 mL flask and heated to 150 °C. The mixture was stirred under vacuum for 30 min. Then, the solution was heated to 260 °C under argon until the mixture turned into transparent. 0.058 g of selenium was dissolved in TOP (0.43 mL) and heated to 200 °C under argon until selenium dissolved, then cooled to room temperature. To achieve the desired particle sizes, selenium

precursor injection temperature and reaction time were optimized. The selenium precursor was injected into the cadmium precursor at 260 °C for CdSe 560 (3 min), at 300 ℃ for CdSe 590 (2 min), and 300 ℃ for CdSe 630 (4.5 min). The yielded CdSe QDs were precipitated twice with ethanol and then dispersed in ODE (2 mL).

OA capped CdSe (CdSe-OA): ODE (5.0 mL), CdO (0.03 g), OA (0.3 mL) were mixed in a 100 mL flask, heated to 150 °C until the mixture turned into transparent. After adding the CdSe/ODE mixture mentioned above, the mixture was heated to 230 °C. After 30 min, OA ligands exchange was achieved. The yielded CdSe QDs were precipitated twice with acetone and then dispersed in toluene.

CdTe: the synthesis of CdTe QDs is according to previous report[2]. CdO (0.013 g), OA (0.14 mL) and ODE (5.0 mL) were mixed in a 50 mL flask and heated to 150 °C. The mixture was stirred under vacuum for 30 min. Then, the solution was heated to 180 °C under argon until the mixture turned transparent. Tellurium (0.064 g) mixed with TBP (0.07 mL) was heated to 200 °C under argon until tellurium dissolved, then cooled to room temperature. The tellurium precursor was injected into the cadmium precursor at 180°C for CdTe 620 (over a period of 1 h). The yielded CdTe QDs were precipitated twice with acetone and then dispersed in toluene.

3 Sample preparation

All samples were prepared in a N_2 glove box to ensure an oxygen-free atmosphere. All the measurement were conducted using a quartz cuvette with a 5 mm path length. QDs, 9-ACA, and motors were directly mixed (toluene as solvent) in the quartz cuvette in the dark at RT.

The concentration of the components (CdSe QDs/9-ACA/motor) for the three color samples are as follows: green 3.1 μM/0.2 mM/0.14 mM; yellow 2.4 μM/0.3 mM/0.38 mM; red 3 μM/0.3 mM/0.22 mM. The QDs/9-ACA ratio was determined by titration, where the concentrated 9-ACA solution was gradually added into the QD solution until the PL quenching of CdSe QDs reached its maximum. Similarly, the concentration of motors was optimized gradually adding concentrated motors solution into the QDs/9-ACA mixture until the highest isomerization efficiency was achieved under visible light irradiation.

4 Spectroscopy

UV-visible absorption spectra were acquired using a Cary 60 UV-visible spectrophotometer from Agilent. Photoluminescence spectra were recorded on a Cary Eclipse fluorescence spectrophotometer (Agilent), and all the spectra were recorded under 400 nm excitation wavelength. UV light irradiation at 365 nm was performed using a UV analytic lamp with an intensity of 16.9 mW/cm^2. Visible light irradiation at 550 nm (140 mW/cm^2), 590 nm (101 mW/cm^2), and 635 nm (280 mW/cm^2) was achieved using LED light sources from Zolix MLED 4-3. Fluorescence lifetime measurements were conducted using time-correlated single photon counting (TCSPC), excited by a 438 nm laser diode from PicoQuant and detected by an MCP-PMT detector, with a recording setting of 10,000 counts across 2048 channels. Circular dichroism (CD) spectra were measured using a Chirascan VX Circular Dichroism Spectrometer from Applied Photophysics. Images of QDs were captured by a field emission scanning electron microscope (TEM, Tecnai G2 F20). Nanosecond time-resolved absorption spectra (nsTA) were recorded on an LP980 transient absorption spectrometer from Edinburgh Instruments, with excitation at 550 nm (21.1 mJ), 580 nm (11.3 mJ), and 600 nm (5.2 mJ). The fsTA measurement was achieved (TA-100, Time-Tech Spectra, Co., Ltd.) by using a Pharos2 fs-laser (Light Conversion; full width at half-maximum, ~222 fs, 100 kHz, 1030 nm), which was split into the pump and probe beams. The pump beam was directed into an optical parametric amplifier (OPA, Orpheus-HP, Light Conversion) to generate a 550 nm excitation (40 nJ per pulse). A frequency-doubled 515 nm light was generated through a β-BaB_2O_4 crystal (BBO crystal), and then passed through a sapphire plate to generate broadband white light (0.3 nJ per pulse, 380 nm to 600 nm). A saturation of the probe beam occurred at 515 nm resulted in a lack of information in this region. An optical delay stage (with maximum delay 6 ns) was used and the two beams were focused and overlapped on the sample in a 1 mm quartz cuvette. The transient spectra and kinetics were acquired by averaging 3,000 times at every given time delay.

5 Size determination of QDs

The diameter (D) of the QDs was determined with empirical formulas and the absorption spectrum using following equation[3].

$$D = (1.6122 \times 10^{-9})\lambda^4 - (2.6575 \times 10^{-6})\lambda^3 + (1.6242 \times 10^{-3})\lambda^2 - (0.4277)\lambda + (41.57)$$

$$\varepsilon = 5857\,(D^{2.65}) \qquad \text{(Eq. S1)}$$

Where, λ (nm) is the first absorption peak wavelength of CdSe QDs (560 nm, 590 nm and 630 nm), and ε ($cm^{-1}M^{-1}$) is molar absorbance coefficient of CdSe QDs at the first absorption peak.

6 Photophysical properties of three batches of CdSe QDs

The photoluminescence quantum yield (ϕ_{PLQY}) of QDs was determined using Rhodamine 6G as a reference ($\phi_r = 0.94$ in ethanol). Rhodamine 6G-ethanol sample was prepared in aerobic environment. In order to avoid inner filter effects, all concentrations were adjusted until the absorbance at the excitation wavelength (500 nm) less than 0.1.

$$\phi_{PLQY} = \phi_r \frac{I\,A_r n^2}{I_r\,A\,n_r^2} \qquad \text{(Eq. S2)}$$

where, *I* is the integrated emission intensity, *A* is the absorption coefficient at the excitation wavelength, and *n* is the refractive index of the solvent. Subscript '*r*' denotes the reference. The photophysical properties of the three batches of QDs are detailed in Table S1.

Table S1 The photophysical properties of three batches of QDs

QDs	Absorption peak (nm)	PL peak (nm)	ϕ_{PLQY} (%)
CdSe 560	560	573	31.2%
CdSe 590	590	610	20.1%
CdSe 630	630	640	10.7%

7 Photoluminescence lifetime of QDs and quenching by 9-ACA

The photoluminescence lifetime decays were deconvoluted with the instrument response function (IRF). The decay fitting of the CdSe and CdSe/9-ACA PL was performed using three exponentials (Eq. S3).

$$I = I_0 \sum_{i=1}^{3} A_i e^{-t/\tau_i} \qquad \text{(Eq. S3)}$$

Where A_i and τ_i are the amplitude and lifetime of the i exponential component. The amplitude-averaged and intensity-averaged lifetimes were respectively calculated with Eq. S4 and Eq. S5.

Amplitude-averaged: $<\tau> = \frac{\sum_{i=1}^{3} A_i \tau_i}{\sum_{i=1}^{3} A_i}$ (Eq. S4)

Intensity-averaged: $\hat{\tau} = \frac{\sum_{i=1}^{3} A_i \tau_i^2}{\sum_{i=1}^{3} A_i \tau_i}$ (Eq. S5)

Table S2. Photoluminescence lifetimes of CdSe and CdSe/9-ACA

Name	CdSe 560	CdSe 560 /9-ACA	CdSe 590	CdSe 590 /9-ACA	CdSe 630	CdSe 630 /9-ACA
**<*τ*> (ns)**	17.1	3.5	20.1	3.3	11.6	1.6
**$\hat{\tau}$ (ns)**	29.3	4.2	28.3	7.2	18.5	2.3

8 Determination of isomer conversion ratio

The concentration of *unstable*-motor under visible light irradiation was determined from the absorption spectra using Lambert-Beer's law. It could be noted that the molar absorption coefficient of the *stable* is negligible at 450 nm. The concentration of *stable* and *unstable* were calculated using Eq. S6.

$$A_{450} = \varepsilon \, C_{unstable} \, L$$
$$C_{stable} = C - C_{unstable} \quad \text{(Eq. S6)}$$

Where, A_{450} isthe change in absorbance at 450 nm after irradiation, ε is the molar absorption coefficient of *unstable* at 450 nm (19 mM^{-1} cm^{-1}), L is the path length of the cuvette (5 mm), $C_{unstable}$ is the concentration of *unstable*-motor after light irradiation, C is the initial concentration of motors before irradiation, and C_{stable} is the concentration of *stable*-motor after irradiation.

9 Photoisomerization quantum yield and conversion

1,2-Bis (2,4-dimethyl-5-phenyl-3-thienyl) perfluoro-cyclopentene (DAE)[4] was used as the actinometer to determine photochemical quantum yields (QY) of the motor under visible light irradiation. The QY was calculated using Eq. S7.

$$\phi = \frac{\Delta A V_1 V_3 N_L}{\Delta t \varepsilon d V_2 I_0} \quad \text{(Eq. S7)}$$

Where, A is optical density of the irradiated solution at irradiation wavelength, t is irradiation time (min), ϕ is quantum yield, ε is extinction coefficient ($mM^{-1}cm^{-1}$), d is the thickness of the cuvette (1 cm), The volumes V_1, V_2, and V_3 represent the irradiated volume, the used volume, and the end volume, respectively. N_L, Avogadro number, I_0, the quantum flow. The absorbance at irradiation wavelengths of all reference samples were exceeded 3, which approximated absorb the entire quantum flow from the light source. For the samples contained the motor sample, the proportion of quantum flow absorbed was determined using Eq. S8,

$$I = (1 - 10^{-A_{CdSe}})I_r \qquad \text{(Eq. S8)}$$

Where, I and I_r are quantum flow absorbed by samples and reference, respectively. A_{CdSe} is the absorbance of CdSe QDs at 550 nm, 590 nm, 620 nm.

Table S3. The Photochemical properties of molecular motors driven under visible light using CdSe

QDs	Drive Wavelength (nm)	*stable*: *unstable* ratio at PSS	ϕ_{QY} (%)
CdSe 560	550	67:33	3.8
CdSe 590	590	69:31	3.3
CdSe 630	635	80:20	2.1

10 Kinetic Analysis of Ultrafast TA Spectroscopy

Exponential growth or decay equations, as detailed in Eq. S9, were employed to analyze the kinetic processes.

$$I = I_0 \sum_{i=1}^{3} A_i e^{-t/\tau_i} \qquad \text{(Eq. S9)}$$

Where, A_i and τ_i are the amplitude and lifetime of the i exponential component. The lifetimes were respectively calculated with Eq. S10.

$$\tau = \frac{\sum_{i=1}^{3} A_i \tau_i^2}{\sum_{i=1}^{3} A_i \tau_i} \qquad \text{(Eq. S10)}$$

The rate constant was determined using Eq. S11, where τ_1 and τ_2 were obtained from fitting the recovery of the CdSe ground state bleaching in the presence and absence of 9-ACA with Eq. S10.

$$k = \frac{1}{\tau} \qquad \text{(Eq. S11)}$$

$$k_{TTET} = \frac{1}{\tau_1} - \frac{1}{\tau_2}$$

Supplemental Figures

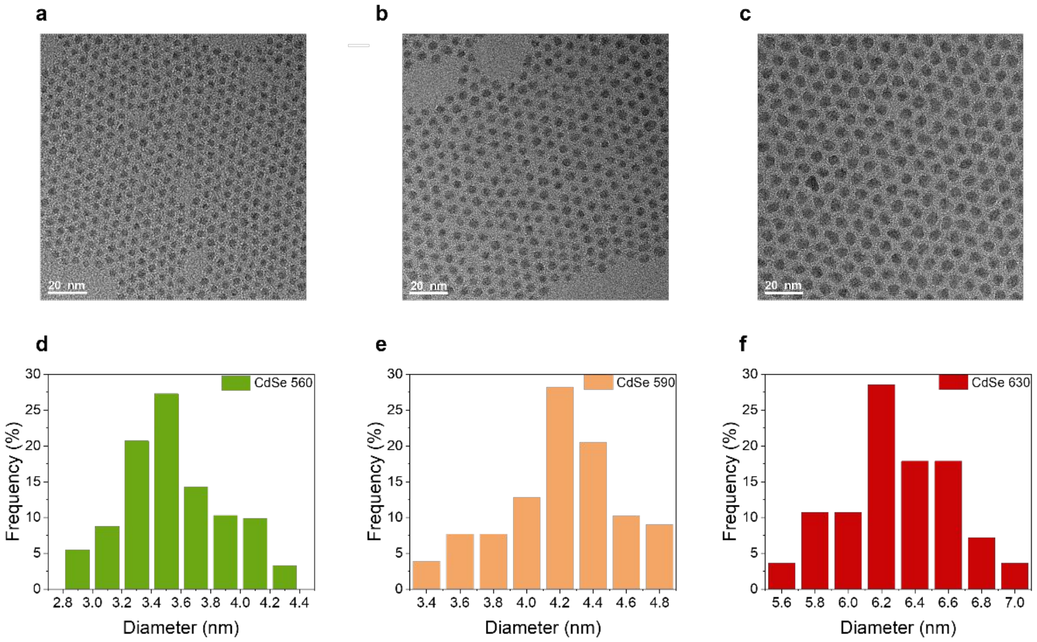

Fig. S1 TEM images of the QDs **a**, CdSe 560, **b**, CdSe 590, **c**, CdSe 630. The scales are located at the bottom left corner of each figure. Size distribution of **d**, CdSe 560, **e**, CdSe590, **f**, CdSe 630 obtained from the statistics of the TEM images.

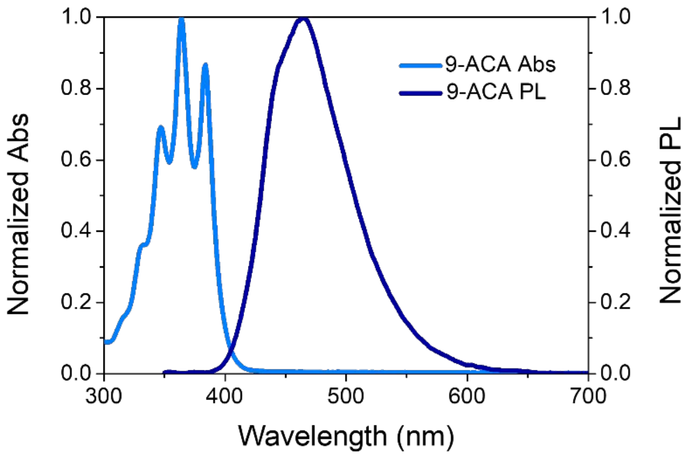

Fig. S2 Normalized absorption and emission spectra of 9-ACA in toluene. The spectra are normalized at 364 nm in absorption and the maximum PL intensity.

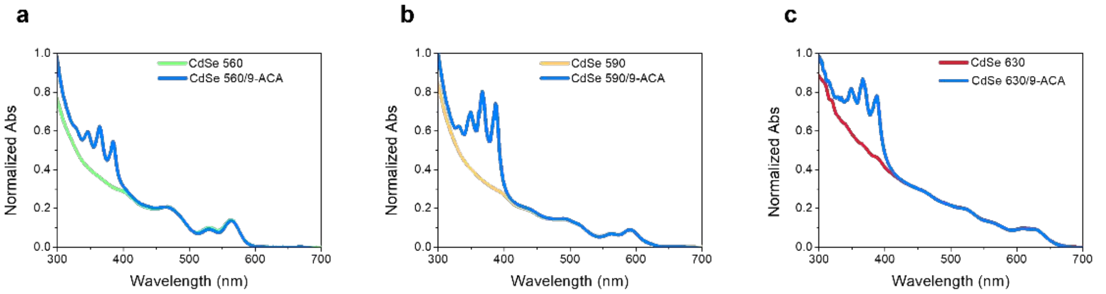

Fig. S3 UV-visible absorption spectra of QDs **a**, CdSe 560, **b**, CdSe 590, **c**, CdSe 630 before and after mixing 9-ACA in toluene.

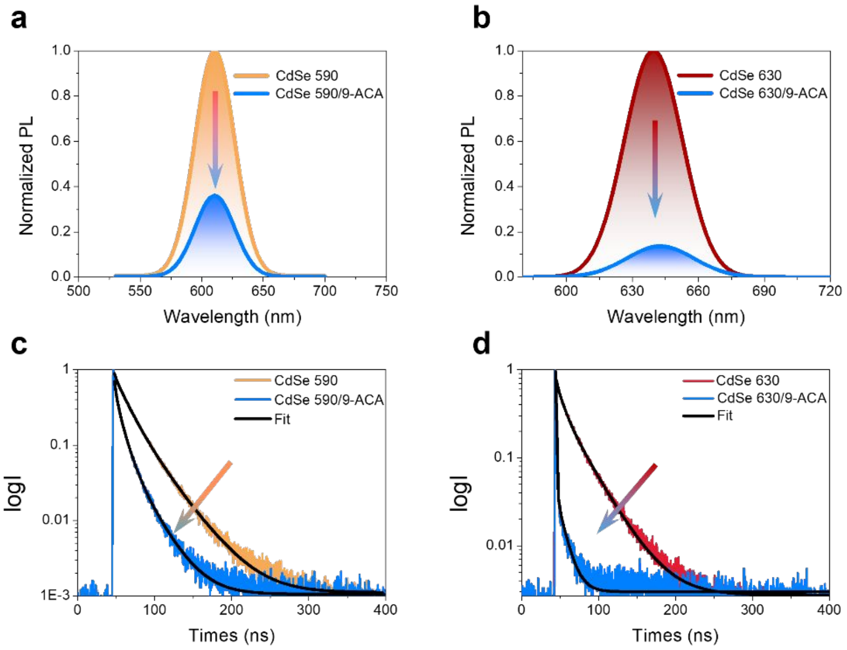

Fig. S4 PL spectra of **a**, 2.4 μM CdSe 590 with 0.3 mM 9-ACA, **b**, 3 μM CdSe 630 with 0.3 mM 9-ACA in toluene. The spectra are normalized at the maximum PL intensity. The PL lifetimes and fitting of **c**, CdSe 590 and **d**, CdSe 630 before and after the addition of 9-ACA. The fitting residuals are shown in Fig. S5

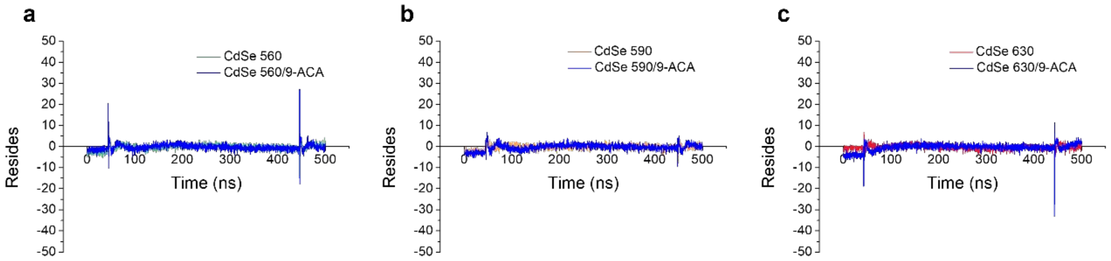

Fig. S5 The corresponding residuals of PL lifetime fitting for CdSe 560 (a), CdSe 590 (b) and CdSe 630 (C). The excitation wavelength was 483 nm for all three samples.

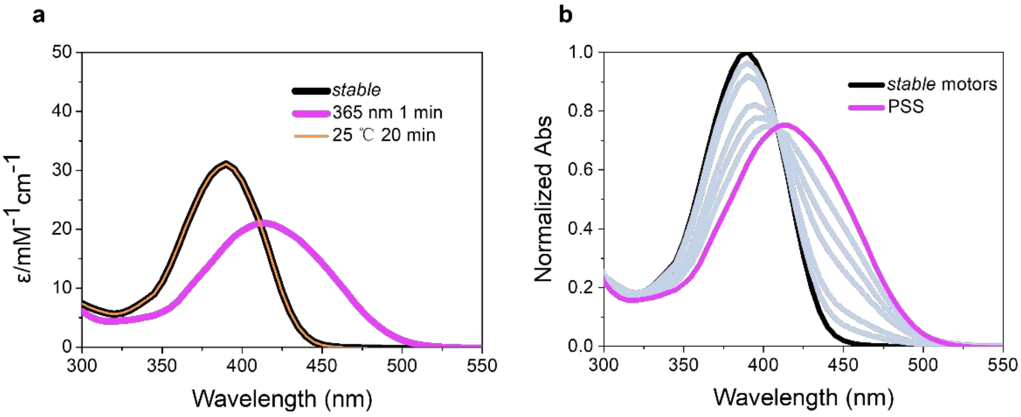

Fig. S6 a, UV-visible absorption spectra of motors in toluene before and after irradiation at 365 nm for 1 min and then remaining in the dark at RT for 20 min. **b**, Formation of the intermediate states under UV irradiation with the isosbestic point identified at 408 nm.

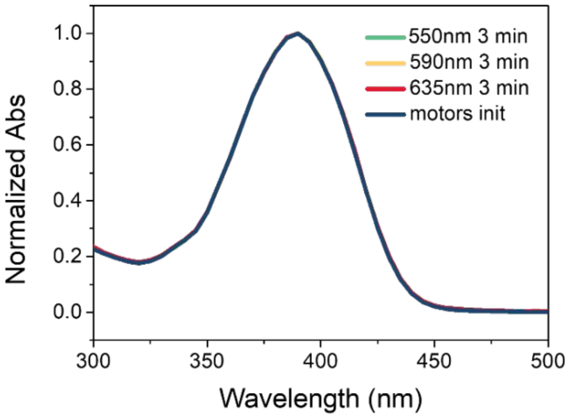

Fig. S7 UV-visible absorption spectra of the motor directly irradiated at 550 nm, 590 nm and 635 nm for 3 min. The spectra are normalized at 390 nm.

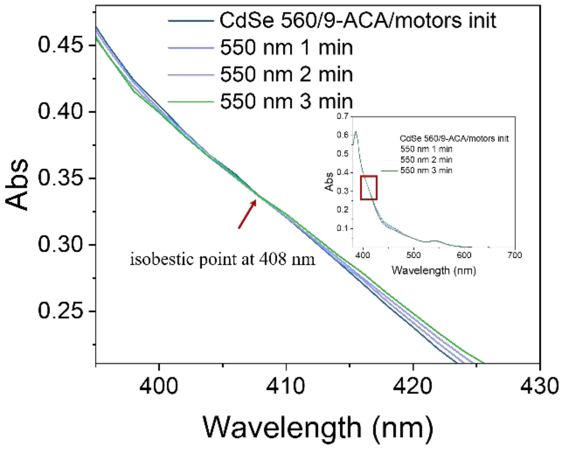

Fig. S8 Indication of the isosbestic point of CdSe 560/9-ACA/motors mixture under 550 nm light irradiation. The insert shows the complete absorption spectra of the sample (380 nm - 700 nm).

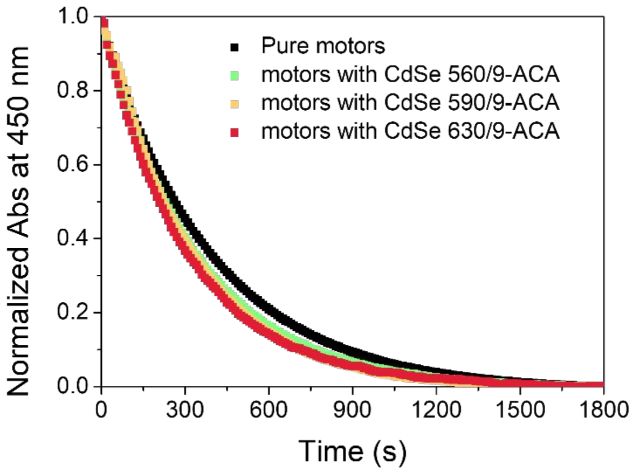

Fig. S9 The THI kinetics of motors mixed with CdSe 560/9-ACA, CdSe 590/9-ACA, and CdSe 630/9-ACA, as well as in the absence of QDs and 9-ACA. The absorption spectra at 450 nm were recorded every 10 second intervals in the dark at RT. The normalization was based on the initial absorbance at 450 nm for each sample.

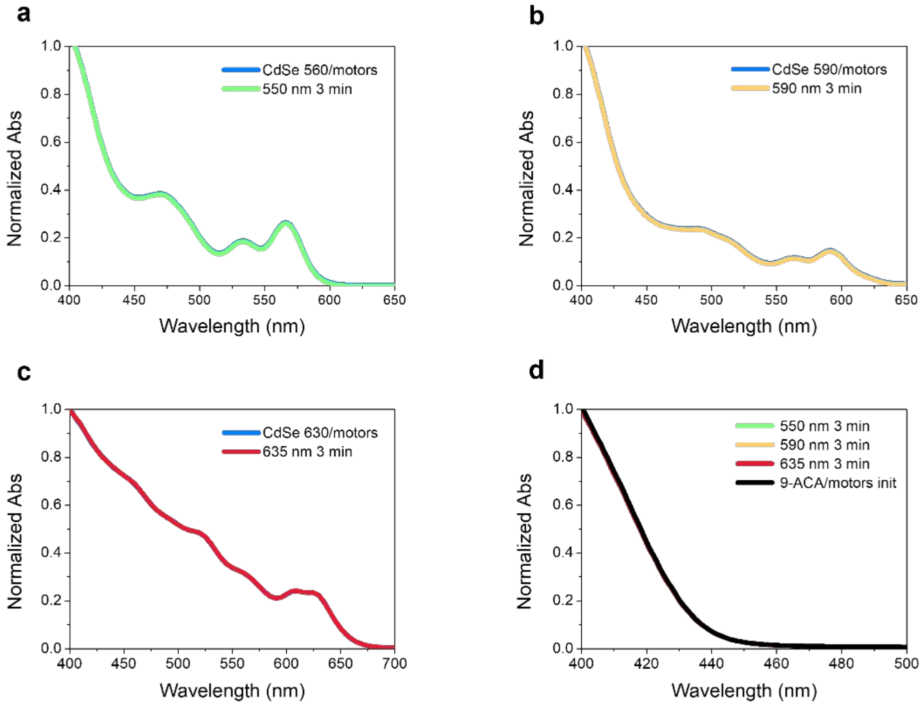

Fig. S10 Mixtures of QDs and motors without 9-ACA under the irradiation at **a**, 550 nm, **b**, 590 nm, **c**, 635 nm (3 min, RT). **d**, Mixtures of 9-ACA and motors without QDs under irradiation at 550 nm/ 590 nm/635 nm (3 min, RT). Spectra are normalized at 400 nm.

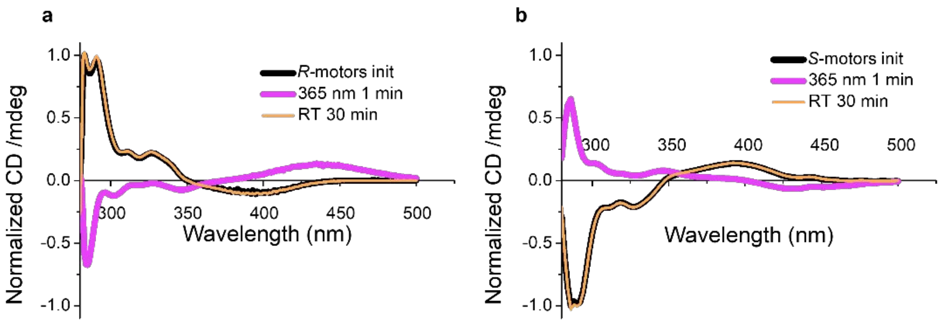

Fig. S11 CD spectroscopy of pure **a,** *R*-motors and **b**, *S*-motors before and after 365 nm irradiation (1min, RT). Subsequent remaining in dark at RT for 30 min. The spectra are normalized at 280 nm.

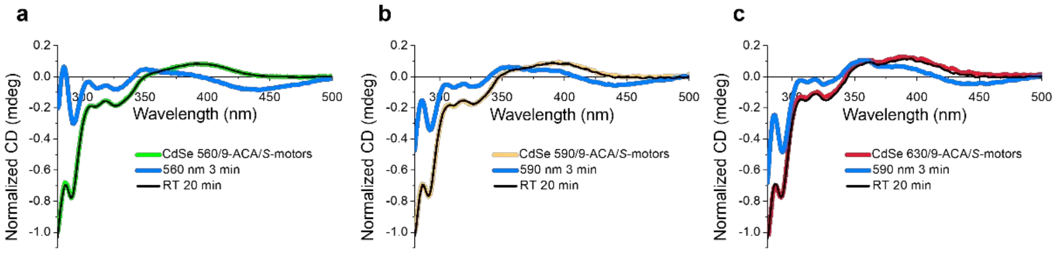

Fig. S12 CD spectra of *S*-motors mixed with **a**, CdSe 560/9-ACA under 550 nm (3 min) light irradiation, **b**, CdSe 590/9-ACA under 590 nm (3 min) light irradiation, and **c**, CdSe 630/9-ACA under 635 nm (3 min) light irradiation. The spectra are normalized at 280 nm.

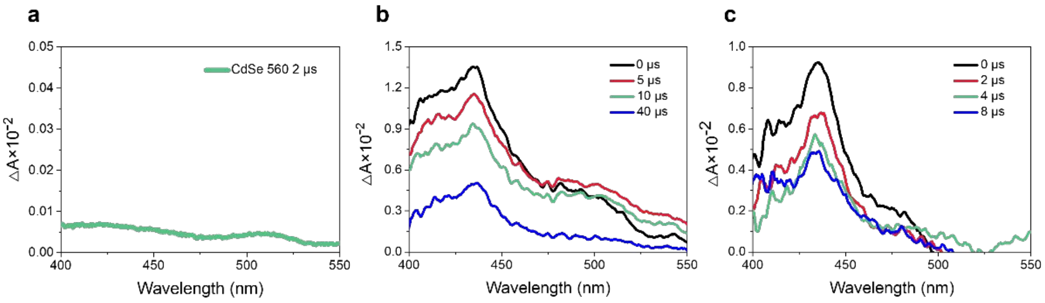

Fig. S13 a, TA spectrum recorded at 2 μs for CdSe 560 alone, **b**, The TA spectra of the mixtures of CdSe 590/9-ACA and **c**, CdSe 630/9-ACA.

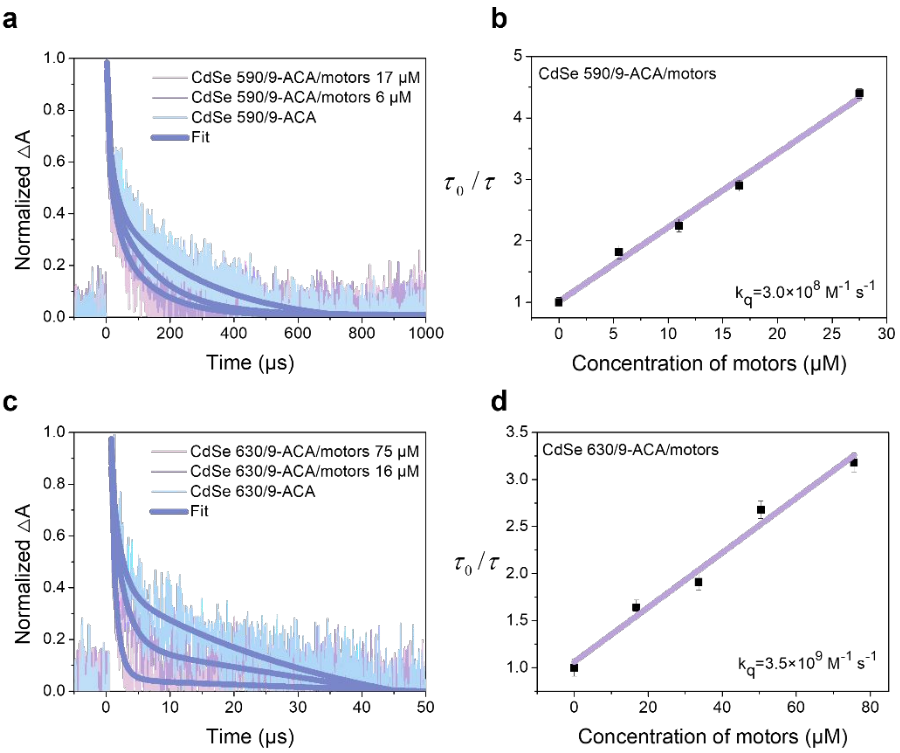

Fig S14. nsTA kinetics at 430 nm of **a**, CdSe 590/9-ACA (under 580 nm pulsed excitation) and **c**, CdSe 630/9-ACA (under 600 nm pulsed excitation) mixed with different concentrated motors. Stern–Volmer plots and linear fits were used to analyze the triplet lifetime quenching of 9-ACA by the motor when mixed with **b**, CdSe 590 and **d**, CdSe 630.

References

(1) Mongin, C.; Garakyaraghi, S.; Razgoniaeva, N.; Zamkov, M.; Castellano, F. N., Direct observation of triplet energy transfer from semiconductor nanocrystals. *Science* **2016**, *351*, 369-372.

(2) Yu, W. W.; Wang, Y. A.; Peng, X., Formation and Stability of Size-, Shape-, and Structure-Controlled CdTe Nanocrystals: Ligand Effects on Monomers and Nanocrystals. *Chem. Mater.* **2003**, *15*, 4300-4308.

(3) Yu, W. W.; Qu, L.; Guo, W.; Peng, X., Experimental Determination of the Extinction Coefficient of CdTe, CdSe, and CdS Nanocrystals. *Chem. Mater.* **2023**, *15*, 2854-2860.

(4) Sumi, T.; Takagi, Y.; Yagi, A.; Morimoto, M.; Irie, M. Photoirradiation wavelength dependence of cycloreversion quantum yields of diarylethenes. *Chem. Commun.* **2014**, *50*, 3928-3930.
